# Supplementary material for: Biosorption of Neodymium (Nd) from Aqueous Solutions Using Spirulina platensis sp. Strains
Source: Polymers (Basel). 2022 Oct 28;14(21):4585. doi: 10.3390/polym14214585 (PMC9654694; doi:10.3390/polym14214585)
Supplement: Supplementary file 1 [file polymers-14-04585-s001.zip › polymers-1936604-supplementary.pdf]

Supplementary Material

2.3 Kinetics, equilibrium, and thermodynamic evaluation

The sorption capacity (Equation S1) and the percentage of Nd(III) removed (Equation S2) are given below:

$$q = \frac{(C_0 - C_f)}{m}.V \qquad \text{(S1)}$$

$$\%\,Removal = 100.\frac{(C_0 - C_f)}{C_0} \qquad \text{(S2)}$$

q is the sorption capacity of Nd(IIII) adsorbed by the adsorbent (mg g^{-1}). C_o is the initial adsorbate solution concentration in contact with the solid adsorbent (mg L^{-1}). C_f is the final adsorbate concentration after adsorption (mg L^{-1}). *m* is the mass of adsorbent (g). *V* is the aliquot of the adsorbate solution (L) introduced in the flask.

The kinetic and equilibrium data's fitness was done using nonlinear methods, which were evaluated using the Simplex method and the Levenberg–Marquardt algorithm using the fitting facilities of the Microcal Origin 2021 software [17,35]. The suitableness of the kinetic and equilibrium models was evaluated using the residual sum of squares (*RSS*), the determination coefficient (R^2), the adjusted determination coefficient (R^2_{adj}), the standard deviation of residues (*SD*), and also the Bayesian Information Criterion (*BIC*) [17,35]. Equations S3 to S7 are the mathematical expressions for respective *RSS*, R^2, R^2_{adj}, *SD, and BIC*.

$$RSS = \sum_{i}^{n} \left(q_{i,exp} - q_{i,model}\right)^2 \qquad \text{(S3)}$$

$$R^2 = \left(\frac{\sum_i^n \left(q_{i,exp} - \overline{q}_{exp}\right)^2 - \sum_i^n \left(q_{i,exp} - q_{i,model}\right)^2}{\sum_i^n \left(q_{i,exp} - \overline{q}_{exp}\right)^2} \right) \qquad \text{(S4)}$$

$$R^2_{adj} = 1 - (1 - R^2).\left(\frac{n-1}{n-p-1}\right) \qquad \text{(S5)}$$

$$SD = \sqrt{\left(\frac{1}{n-p}\right) \cdot \sum_{i}^{n} \left(q_{i,exp} - q_{i,model}\right)^2} \quad (S6)$$

$$BIC = nLn\left(\frac{RSS}{n}\right) + pLn(n) \quad (S7)$$

In the above equations, $q_{i,\ model}$ is the individual theoretical q value predicted by the model; $q_{i,\ exp}$ is individual experimental q value; $\overline{q}_{exp}$ is the average of all experimental q values measured; n is the number of experiments; p is the number of parameters in the fitting model.

The values of R^2_{adj}, *SD*, and *BIC* will be presented to compare different models of kinetics and equilibrium presented in this work. The best-fitted model would present R^2_{adj} closer to 1.000, lower values of SD, and BIC values. However, the kinetic and equilibrium model could not merely be chosen based on the values of R^2 [17,35] when these models present a different number of parameters. Therefore, it is necessary to check if the R^2 values' improvements are due to the increase of a number of the parameters [17,35] or if, physically, the model with more parameters explains better the process taking place [17,35].

However, the difference in BIC values between models could be conclusive if the difference in BIC values ≤ 2.0) shows no significant difference between the two models [17,35]. When BIC values' difference is within 2-6, there is a positive perspective that the model with lower BIC is the most suitable [17,35]. For variations of BIC values from 6-10, there is a strong possibility that the model with a lower BIC value is the best model to be fitted [17,35]. However, if the difference in BIC values ≥ 10.0, it can be predicted with accuracy that the model with a lower BIC value is better fitted [35,36].

Pseudo-first-order [17,35], pseudo-second-order [17,35], and Avrami fractional-order [17] models were used to fit the kinetic data. The mathematical equations of these respective models are shown in Equations S8, S9, and S10.

$$q_t = q_e.[1\text{-}exp(\text{-}k_1 t)] \quad \text{(S8)}$$

$$q_t = \frac{k_2.q_e^2.t}{1 + q_e.k_2.t} \quad \text{(S9)}$$

$$q_t = q_e.[1 - exp\ (-k_{AV}.t)^{nAV}] \quad \text{(S10)}$$

Where t is the contact time (min); q_t, and q_e are the amount of adsorbate adsorbed at time t and the equilibrium, respectively (mg g^{-1}); k_1 is the pseudo-first-order rate constant (min^{-1}); k_2 is the pseudo-second-order rate constant (g mg^{-1} min^{-1}); k_{AV} is the Avrami-fractional-order constant rate (min^{-1}), n_{AV} is the Avrami exponent (n >0).

Langmuir, Freundlich, and Liu's models were employed to analyze equilibrium data. Equations S11, S12 and S13 show the corresponding Langmuir [17], Freundlich [17], and Liu models [17].

$$q_e = \frac{Q_{max}.K_L.C_e}{1 + K_L.C_e} \quad \text{(S11)}$$

$$q_e = K_F.C_e^{1/nF} \quad \text{(S12)}$$

$$q_e = \frac{Q_{max}.(K_g.C_e)^{nL}}{1 + (K_g.C_e)^{nL}} \quad \text{(S13)}$$

Where q_e is the adsorbate amount adsorbed at equilibrium (mg g^{-1}); C_e is the adsorbate concentration at equilibrium (mg L^{-1}); Q_{max} is the maximum sorption capacity of the adsorbent (mg g^{-1}); K_L is the Langmuir equilibrium constant (L mg^{-1}); K_F is the Freundlich equilibrium constant [mg.g^{-1}.(mg.L^{-1})$^{-1/nF}$]; K_g is the Liu equilibrium constant (L mg^{-1}); n_F and n_L are the exponents of Freundlich and Liu model, respectively, (n_F and n_L are dimensionless).

Thermodynamic studies for the Nd(III) adsorption onto LEB-18 and LEB-52 *Spirulina platensis* algae adsorbents were performed at temperatures ranging from 22ºC to 55°C (298 to 328 K).

The Gibb's free energy change (ΔG^0, kJ mol^{-1}), enthalpy change (ΔH^0, kJ mol^{-1}), and entropy change (ΔS^0, J $mol^{-1}K^{-1}$) were evaluated with the aid of Equations S14–S17,

respectively [17,36].

$$\Delta G^0 = \Delta H^0 - T.\Delta S^0 \quad \text{(S14)}$$

$$\Delta G^0 = -RT.LnK_e^0 \quad \text{(S15)}$$

$$K_e^0 = \frac{(1000.K_g.Mw.[adsorbate]^0}{\gamma} \quad \text{(S16)}$$

The combination of Equations S14 and S15 leads to Equation S17

$$LnK_e^0 = \frac{\Delta S^0}{R} - \frac{\Delta H^0}{R}.\frac{1}{T} \quad \text{(S17)}$$

R is the universal gas constant (8.314 J K^{-1} mol^{-1}); T is the absolute temperature (Kelvin); M_w is the molecular weight of the adsorbate (g mol^{-1}), $[adsorbate]^0$ is the standard molar concentration of the adsorbate, which by definition is 1 mol L^{-1}; γ is the activity coefficient of the adsorbate. K_e^0 is the thermodynamic equilibrium constant, calculated according to Equation S16. K_e^0 is dimensionless [17,36].

K_e^0 is calculated by converting K_g values (Liu equilibrium constant) or K_L (Langmuir equilibrium constant), expressed in L mg^{-1} into L mol^{-1}. Firstly, the value K_g or K_L is multiplied by 1000 (mg g^{-1}), and then multiplied by the molecular weight of the adsorbate (g mol^{-1}) and by the standard concentration of the adsorbate (1 mol L^{-1}) and divided by the activity coefficient of the adsorbate (γ- dimensionless) [17,36]. It is assumed that the solution is sufficiently diluted to consider that the γ is unitary [17,36]. Making these calculations, K_e^0 becomes dimensionless [17,36].

Equation S17 is the linearized van't Hoff equation [17]. On the other hand, Lima et al. [17] recently proposed using the nonlinear van't Hoff equation, as presented in Equation S18.

$$K_e^0 = exp\left[\frac{\Delta S^0}{R} - \left(\frac{\Delta H^0}{R}\right).\frac{1}{T}\right] \quad \text{(S18)}$$
